# Pigmented Epithelioid Melanocytoma (PEM)/Animal Type Melanoma (ATM): Quest for an Origin. Report of One Unusual Case Indicating Follicular Origin and Another Arising in an Intradermal Nevus †

**DOI:** 10.3390/ijms18081769

**Published:** 2017-08-15

**Authors:** Ashley Tarasen, J. Andrew Carlson, M. Kathryn Leonard, Glenn Merlino, David Kaetzel, Andrzej T. Slominski

**Affiliations:** 1 Departments of Dermatology and Pathology, University of Alabama at Birmingham, Birmingham, AL 35201, USA; ashleytarasen@gmail.com; 2 Department of Dermatopathology, Albany Medical College, Albany, NY 12201, USA; carlsoa@mail.amc.edu; 3 Department of Biochemistry and Molecular Biology, Marlene and Stewart Greenebaum Cancer Center, University of Maryland School of Medicine, Baltimore, MD 21201, USA; KLeonard@som.umaryland.edu (M.K.L.); DKaetzel@som.umaryland.edu (D.K.); 4 Center for Cancer Research, National Cancer Institute, Bethesda, MD 21201, USA; gmerlino@helix.nih.gov; 5 Veterans Affairs Medical Center, Birmingham, AL 35201, USA

**Keywords:** melanocytes, melanoma, hair follicle, hair follicle stem cell, melanin pigment

## Abstract

Pigmented epithelioid melanocytoma (PEM) is a tumor encompassing epithelioid blue nevus of Carney complex (EBN of CNC) and was previously termed animal-type melanoma. Histologically PEMs are heavily pigmented spindled and epithelioid dermal melanocytic tumors with infiltrative borders, however, their origin remains unclear. Stem cells for the epidermis and hair follicle are located in the bulge area of the hair follicle with the potential to differentiate into multiple lineages. Multiple cutaneous carcinomas, including follicular cutaneous squamous cell carcinoma (FSCC), are thought to arise from stem cells in the follicular bulge. We present two cases of PEM/ATM in a 63 year-old male on the scalp with follicular origin and a 72 year-old female on the upper back arising in an intradermal nevus. Biopsy of both cases revealed a proliferation of heavily pigmented dermal nests of melanocytes with atypia. The Case 1 tumor was in continuation with the outer root sheath of the hair follicle in the bulge region. Case 2 arose in an intradermal melanocytic nevus. Rare mitotic figures, including atypical mitotic figures, were identified in both cases. We present two cases of PEM, with histologic evidence suggesting two origins: one from the follicular bulb and one from an intradermal nevus.

## 1. Introduction

Pigmented epithelioid melanocytoma (PEM), also known as pigment synthesizing melanoma [1,2,3,4], is an entity encompassing epithelioid blue nevus (of Carney complex) and previously termed animal-type melanoma (ATM) [5,6]. PEMs show a striking histological similarity to ATM [7,8,9] and epithelioid blue nevi [10,11,12]. The term ATM was used because of their striking similarity in morphology with experimental models of melanomas in mice or horses [8,9,13,14].

Studies comparing PEM and epithelioid blue nevi have been unable to find histologic criteria separating metastasizing from nonmetastasizing PEMs [6]. Zembowicz identified no correlation between the presence of ulceration, degree of cytologic atypia or mitotic activity, and the finding of lymph node metastasis. Even more disconcerting was that their study found metastases occurring in bland lesions with no mitotic activity [6]. Although PEM has been reported to frequently be found in sentinel lymph nodes (SLNs), a short-term follow-up suggests a better prognosis than conventional melanoma [6]. Histologically, PEM is described as a heavily pigmented tumor composed of spindled and epithelioid melanocytes with infiltrative borders [6]. The tumor may either abut the epidermis or be separated by a Grenz zone [6]. The origin of these tumors remains unclear [6]. Herein, we present two cases of PEM/ATM with histology suggesting a possible origin in the follicular bulge and an intradermal nevus. The hypothesis of follicular origin is also discussed in the context of melanoma origin in rodents.

## 2. Case Reports

The use of the archival material was approved by the Institutional Review Board at the University of Alabama at Birmingham under exempt category #4 (IRB registration No. IRB00000726).

### 2.1. Case 1

**Figure 1:**
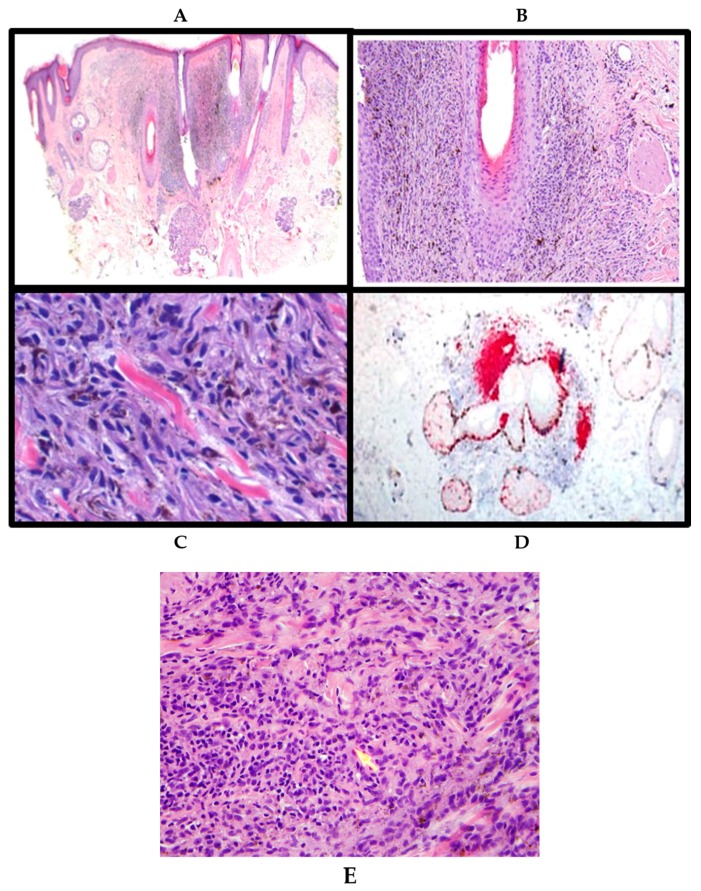
Punch biopsy revealed a tumor located predominantly in the dermis (**A**); The tumor cells were composed of spindled, dendritic, and epithelioid melanocytes containing variable amounts of melanin pigment, with some melanocytes being very heavily pigmented, mcrospoic magnification (mm): ×4 (**B**); Multiple level sections demonstrated that the tumor cells were growing in sheets originating in continuation with nests in the hair follicle, mm: ×10 (**C**); The tumor cells involved the follicular structures in nests or groups of atypical melanocytes growing in the outer root sheath of hair follicles, involving sebaceous glands, with predominant accumulation in the bulge area (red stain as identified by antibodies against MART-1 and SOX-10 antigens), mm: ×20 (**D**); Mitotic figures were identified (arrow), mm: ×10 (**E**).

A 63-year-old male was presented to surgical oncology clinic for a scalp lesion that was worrisome for malignancy. The lesion was identified on SPOTme^®^ skin cancer screening [15]. The patient spends a significant amount of time outside in the sun, playing tennis, and does not wear sunscreen. Physical exam revealed a dermal-based nodule on the scalp with blue pigmentation. Punch biopsy revealed a tumor located predominantly in the dermis (Figure 1A). The tumor cells were composed of spindled, dendritic, and epithelioid melanocytes containing variable amounts of melanin pigment, with some melanocytes being very heavily pigmented (Figure 1B). Multiple level sections demonstrated that the tumor cells were growing in sheets originating in continuation with nests in the hair follicle (Figure 1C). The tumor cells involved the follicular structures in nests or groups of atypical melanocytes growing in the outer root sheath of hair follicles, involving sebaceous glands, with predominant accumulation in the bulge area (as identified by antibodies against MART-1 and SOX-10 antigens) (Figure 1D). Tumor invasion of smooth muscle bundles was identified. Occasional mitotic figures with one atypical mitotic figure were identified (Figure 1E). The maximum tumor thickness was 1.4 mm with Clark’s level IV. The tumor sections were sent for fluorescent in situ hybridization (FISH) analysis, however the amount of diagnostically relevant tissue was insufficient for a definitive analysis in the later samples. The histologic findings were consistent with a PEM/ATM with follicular origin. This patient was unavailable for a follow-up.

### 2.2. Case 2

**Figure 2:**
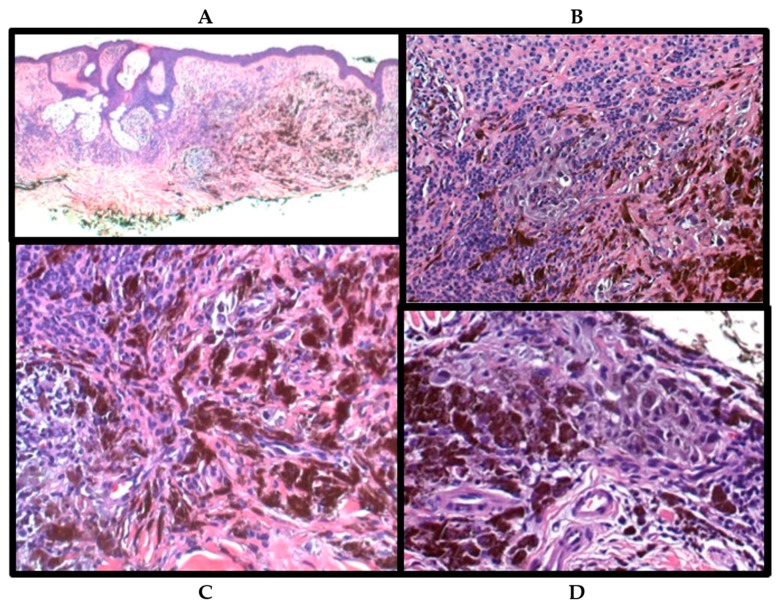
Shave biopsy of dermal melanocytic proliferation arising in the background of an intradermal melanocytic nevus (**A**,**B**). PEM arising in the intradermal nevus (**B**–**D**). The sections were stained with eosin and heamatoxylin (H&E). Brown color represent melanin pigment. Microscopic magnification for (**A**): ×4, for (**B**): ×10, for (**C**,**D**): ×20.

A 72-year-old male was presented for an annual skin check. A soft brown papule with hyperpigmentation on the inferior edge was identified on his back. The clinical differential diagnosis was a nodular malignant melanoma versus a melanocytic nevus. Shave biopsy revealed a heavily pigmented spindle, dendritic, and epithelioid melanocytic proliferation in the dermis arising in the background of an intradermal melanocytic nevus, congenital pattern (Figure 2A,B). At least two mitotic figures were identified. Level sections revealed multiple areas of the PEM arising in and from the intradermal nevus (Figure 2B–D). The patient later underwent an excision, which was negative for residual melanocytic tumor, and a sentinel lymph node biopsy in which three sentinel lymph nodes were negative for melanoma (0/3). The patient is tumor free at six months follow-up.

## 3. Melanomas in Rodents

**Figure 3:**
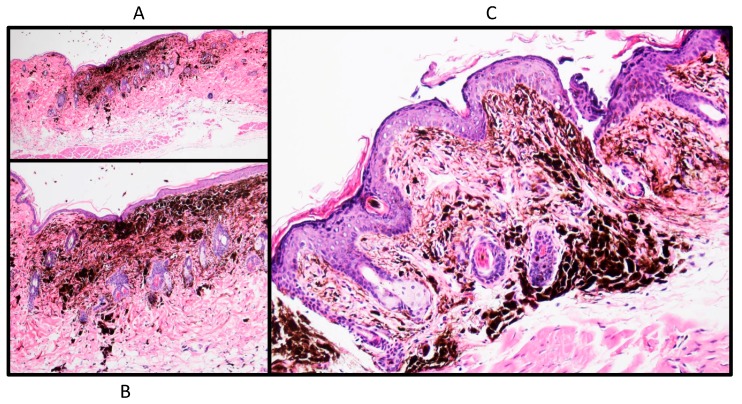
Analysis of a typical UV-induced melanoma obtained in the HGF/SF (Hepatocyte growth factor/scatter factor)-overexpression model (Noonan, 2000). Mice were irradiated at post-natal day four with 4 kJ/m^2^ of ultraviolet light (UV) (61% UVB, 28.3% UVA, 2.09% UVC). Once tumors were palpable, the animals were sacrificed and the skin with tumors fixed and processed as described previously [20]. The sections were stained with H&E. Brown color represent melanin pigment. Microscopic magnification for (**A**): ×4, for (**B**): ×10, for (**C**): ×40.

The morphology of the above tumors is strikingly similar to melanomas in rodents [16,17,18]. For example, we have observed that mouse melanomas that were obtained following exposure to ultraviolet light (UVR) engineered for overexpression of hepatocyte growth factor/scatter factor (HGF/SF) [19] and were further genetically modified for reduced expression of the metastasis suppressor NM23-H1 [16,20] typically display a proliferation of dendritic or spindled heavily pigmented melanocytes with epithelioid cells in the dermis and infiltrative borders [20]. At the early stages these lesions resemble blue nevi [20] that later develop into heavily pigmented tumors with dendritic, spindle, and some epithelioid cells invading the dermis and adjacent structures (Figure 3). Despite the benign looking morphology on the initial stages [20] and rather borderline morphology of developed lesions (Figure 3), the tumors metastasize and kill the animals [16,19].

## 4. Discussion

PEM is an entity that encompasses epithelioid blue nevus of Carney complex (EBN of CNC) and most lesions earlier considered to be labelled ATM or pigment synthesizing melanoma [2]. The concept of ATM is not widely accepted in humans [6] due to the lack of definitive histologic criteria distinguishing benign from metastasizing melanocytic tumors. PEM may frequently show a loss of expression of a gene mutated in families with CNC, a cyclic AMP (adenosine monophosphate)-dependent protein kinase 1 R1α, supporting that it is a distinct tumor [21]. Loss of R1α expression helps explain the dark pigmentation of the tumor [5]. Heavily pigmented melanocytic tumors are a diagnostic challenge due to some behaving in an indolent fashion (EBN of CNC) and others, being morphologically indistinguishable, having aggressive behavior and termed ”animal-type melanoma” [22]. Therefore, subsequent terminology of PEM was suggested with sentinel lymph node biopsy as a “diagnostic procedure” to better characterize the biologic behavior of these tumors [6]. Reports show that PEM are frequently found in sentinel lymph nodes (SLN’s) but short-term follow-up suggests a better prognosis for PEM than for conventional metastatic melanoma [6,23,24]. It has been proposed that PEM be classified as a borderline tumor or a low-grade melanoma with potential lymph node metastasis but a less frequent systemic spread [6]. The origin of these tumors remains unclear.

Evidence from the literature has documented that stem cells for the epidermis and hair follicle are located in the bulge area of the hair follicle [25]. These cells have the potential to differentiate into multiple cell lineages including epidermal and follicular keratinocytes, epidermal and follicular melanocytes, and sebocytes [26,27], with melanocytic differentiation regulated by hormonal, nutritional, and local trophic factors [28,29,30,31,32,33]. Follicular stem cells likely represent the origin of rodent (at least mouse) melanomas, since in adolescent and adult mice corporal skin melanocytes are confined to the hair follicle [34], and melanogenesis is coupled to anagen phase of hair growth [35]. The hair bulb is the only site of pigment production in the corporal skin of adult mice to form a pigmented hair shaft [27,34,36,37,38,39,40,41]. Spontaneous or induced melanomas in laboratory rodents and nocturnal animals is very rare and either originate from intradermal melanocytic nevi in hamsters or from follicular melanocytes, or precursors to them, in mice or in genetically modified mice [17,42,43,44]. The presented histology on murine melanomas is strikingly similar to the presented cases of PEM (Case 1). During induction of anagen, melanoblasts in the bulge region are induced to migrate along the outer route sheath gradually gaining melanocyte differentiated functions on its way to the location in hair matrix above the dermal papilla, where they become melanogenically active [27,34]. These migrating and differentiating follicular melanocyte stem cells will be vulnerable to chemical, biological, or physical insult that may change the normal pathway leading to malignant transformation. Therefore, we propose that such pigmented melanocytic tumors, such as PEM, may arise from the stem cells localized in the follicular bulge, as demonstrated on histologic sections of Case 1. It is generally recognized that cutaneous squamous cell carcinoma (SCC) arises from the epidermis and not the adnexal structures. However, a recently described subtype of cutaneous SCC: follicular squamous cell carcinoma (FSCC), is defined as an SCC arising in the wall of a hair follicle without a demonstrable epidermal point of origin [45,46,47,48,49,50,51,52,53]. These have been reported to occur on the head and upper extremities of elderly people [45,53] and behave poorly as aggressive tumors [53]. Other examples of malignant hair follicle tumors include the trichilemmal carcinoma, trichoblastic carcinoma, pilomatrixal carcinoma, sebaceous carcinoma, and malignant proliferating trichilemmal cyst [7].

## 5. Conclusions

Most cutaneous malignancies occur and arise within the epidermis and are usually attributed, at least in part, to UV damage. Until now, the origin of PEM was unknown. We present one case with evidence to support that PEMs possibly arise from the bulge region of hair follicles, where the cutaneous stem cells are located, and another arising in an intradermal nevus.
